# Carlow Virus, a 2002 GII.4 variant Norovirus strain from Ireland

**DOI:** 10.1186/1743-422X-4-61

**Published:** 2007-06-13

**Authors:** Karen Kearney, John Menton, John G Morgan

**Affiliations:** 1 Lab 439, Department of Microbiology, University College Cork, College Road, Cork, Ireland

## Abstract

**Background:**

Noroviruses are the leading cause of infectious non-bacterial gastroenteritis in Ireland (population 4 million). Due to the number of outbreaks, its massive impact on the Irish health service and its seasonality, Norovirus has gained public notoriety as The Winter Vomiting Bug. The increase in cases in Ireland in the 2002–2003 season coincided with the emergence of two new Genogroup II genotype 4 variant clusters of Norovirus worldwide.

**Results:**

Little research has been done on the epidemiology or molecular biology of Norovirus strains in Ireland. In an effort to combat this discrepancy, we cloned a full length human norovirus genome as a cDNA clone (J3) which can produce full length transcripts in vitro. A polymerase mutant cDNA clone (X1), in addition to a sub genomic cDNA clone (1A) were produced for use in future work.

Carlow virus (Hu/NoV/GII/Carlow/2002/Ire) genome is 7559 nts in length, excluding the 3-end poly A tail and represents the first Norovirus strain from Ireland to be sequenced.

**Conclusion:**

Carlow virus is a member of the Farmington Hills variant cluster of Genogroup II genotype 4 noroviruses.

## Background

Noroviruses are the leading cause of infectious non-bacterial gastroenteritis in Ireland.

The Department of Health, reported 7,500 cases of suspected Norovirus infection in the 2002 season. The National Disease Surveillance Centre stated that the majority of these outbreaks occurred in a health care setting with significant associated morbidity. Recent studies are now implicating Norovirus as a cause of death, a fact that has frequently been masked by the very location of the outbreaks and their residents, namely nursing homes and the elderly [1].

Norovirus is a member of the *Caliciviridae *family of viruses. It is a single stranded, positive sense, RNA virus of 7.4–7.7 kb in length, with a 3' poly A tail. The genome is organised into three Open Reading Frames (ORFs). ORF1 encodes an approximate 200 kDa polyprotein, which is proteolytically processed into the N terminal protein, NTPase, p22, p20, VpG, 3C-like protease and RNA dependent RNA polymerase [2]. ORF2 and 3 encode the structural proteins, capsid VP1 and minor structural protein VP2. The capsid is divided into two domains the N-terminal shell (S) and the C-terminal protusion (P), linked by an eight amino acid hinge. The P domain consists of the P1 and P2 subdomains. The P2 domain located on the surface of the capsid binds the histo-blood group antigens [3,4].

Noroviruses are assorted into five Genogroups based on sequence identity. Genogroup I, II and IV are associated with human norovirus infection. Norovirus strains can be further divided based on sequence homologies into 14 GI and 17 GII genotypes [5]. GII.4 noroviruses are the predominant circulating genotype worldwide [6].

The increase in cases in Ireland in the 2002–2003 season, coincided with the emergence of two new Genogroup II genotype 4 variant clusters of Norovirus. These viruses were part of a global epidemic that occurred in 1995 (US 95/96) and 2000 (Farmington Hills) [6]. These variant viruses arise as a consequence of Norovirus being highly adaptive, harbouring an RNA dependent RNA polymerase, without proof reading capabilities, which allows a high rate of mutations to accumulate, with many apparent within the capsid receptor binding region. These mutations may provide an opportunity to escape host immunity [7,8,1].

Noroviruses are known to bind to the blood group antigens in a strain specific manner [9]. The histo-blood group antigens are highly polymorphic, a characteristic, which may assist host immunity from infection, as these complex carbohydrates also act as receptors for other organisms such as *H. pylori *and *E. coli *R45 [10,11]. They are present on the surfaces of red blood cells and on the surface of the mucosal epithelium of the respiratory, genitourinary and digestive tracts. They also exist as free oligosaccharides, in biological fluids, such as saliva, intestinal contents, milk and blood [[12-14] for review].

The heterogeneity of the blood group antigens in conjunction with the diversity of Norovirus strains has hampered receptor binding studies due to the vast number of possible binding patterns. Advances have been made in the study of human norovirus biology such as the development of an in vitro replication system in a cell line [15-17]. In addition, the propagation of murine norovirus in cell culture has been a major accomplishment providing a novel animal model [18].

Research on the epidemiology or molecular biology of Norovirus strains in Ireland is at a very preliminary stage. In an effort to overcome this we firstly screened 70 stool samples from five different outbreaks that occurred in a hospital setting to identify the most prevalent circulating genotype. A representative of this genotype was then cloned as a full length human norovirus genome, denoted Carlow virus. Comparative sequence analysis was performed using this virus against other noroviruses in the database [GenBank] in order to identify differences, if any, between Carlow virus and other viruses circulating prior to the 2002 season. In addition, a polymerase mutant genome and a representative clone of the sub genomic RNA as cDNA clones were generated for use in future work.

## Results

### Screening

70 stool samples were obtained from five different hospital outbreaks in the south eastern region of the country. Screening of the 70 samples utilising COG2F [19] and the reverse primer G2NVR designed to the 3' end of ORF1 and start of ORF2 (Table 1) showed that 32 samples were positive for Norovirus. 28 were found to be members of Genogroup II (GII), 2 of Genogroup I (GI) and 2 not yet determined. The Genogroup I isolates belong to Genotype 1 and 3, respectively. Six of the 28 strains were sequenced and were shown to be members of the Genogroup II genotype 4 group of noroviruses. One such strain was cloned in this study after only one round of RT-PCR, in conjunction with restriction endonuclease methodologies, as a full length cDNA clone (J3) into pBluescript II SK+ and was denoted Carlow virus.

**Table 1:** 

**Primer**	**Sequence**	**Polarity**	**Ref**
JV12	ATACCACTATGATGCAGATTA (4279–4299)	+	[28]
SM31	CGATTTCATCATCACCATA (4592–4610)	-	[28]
COG2F	CARGARBCNATGTTYAGRTGGATGAG (5003–5028)	+	[19]
G2NVR	ACCNGCATANCCRTTRTACATTC (5365–5387)	+	
TX30SXN	GACTAGTTCTAGATCGCGAGCGGCCGCCC (T × 30)	-	[29]
ORF3minusdegen	ATCTCCTTRTCATGWTTRAARGAAGCC (6868–6894)	-	
430F	ATGTGGGAYGGRGAGATCTAC (398–418)	+	
4440 minus	TCGTTGATTGATATTGTGAAGTC (4436–4458)	-	
4440 nest	TTGATTGATATTGTGAAGTC (4436–4455)	-	
5090 R	TCATTCGACGCCATCTTCATT (5084–5104)	-	
4290 F	TCACTATGATGCTGATTACTC (4282–4302)	-	
NLV1S25F	GTGAATGAAGATGGCGTCTAACGAC (1–25)	+	
NEWRACE	ATAGCAATTGTTGTCAAAGGCTGTGTAAGGGAACG (588–622)	-	

### The genome

The Carlow virus (Hu/NoV/GII/Carlow/2002/Ire) genome is 7559 nts in length, excluding the 3' poly A tail [GenBank: DQ415279]. There are three ORFs, (ORF1, nt 5–5104; ORF2, nt 5085–6707; and ORF3, nt 6707–7513) which harbour the potential to encode the non structural polyprotein and the structural proteins, capsid VP1 and minor structural protein, VP2. Blastn alignments of the entire genome of Carlow virus revealed 99% identity to the Norovirus GII genotype 4 variant members HU/NoV/Farmington Hills/2002/USA [GenBank: AY502023] and the United Kingdom strain B4S6 [GenBank: AY587985], whereas only 90% identity was observed to the prototype GII.4 member, Lordsdale virus [GenBank: X86557].

Blastp alignments utilising the potential amino acid sequences of all three ORFs were performed. The putative polyprotein of Carlow virus (1700 a.a) exhibits 99% identity to a number of strains including HU/NoV/Farmington Hills/2002/USA [GenBank: AY502023], Hu/NoV/CS G12002/USA [GenBank: AY502020.1] and Hu/NLV/Oxford/B4S2/2002/UK [GenBank: AAT00233.1]. Carlow virus showed 96% identity to the equivalent protein of Lordsdale virus [GenBank: CAA60254].

The potential capsid protein of Carlow virus conveys 99% identity to a collection of strains such as Hu/NoV/Germanton/2002/USA [GenBank: AAR97645.1], Hu/NLV/Oxford/B4S2/2002/UK [GenBank: AAT00234.1], Hu/NoV/Farmington Hills/2002/USA [GenBank: AAR97663.1] and NLV/GII/Langen1061/2002/DE [Genbank: AAR32988.1]. 93% identity was observed to the capsid of Lordsdale virus [GenBank: CAA60255].

VP2 exhibits 98% to a variety of strains which includes Hu/NLV/Oxford/B4S2/2002/UK [GenBank: AAT00235.1] and Hu/NoV/Farmington Hills/2002/USA [GenBank: AY502023.1]. 86% identity was observed to VP2 of Lordsdale virus [GenBank: CAA60256].

**Figure 1:**
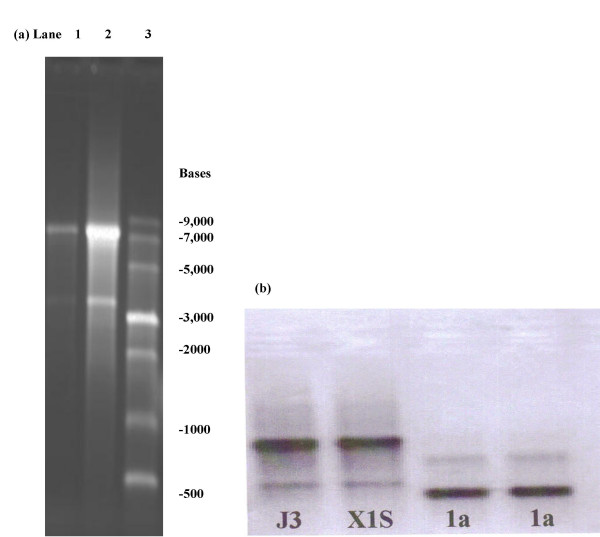
(a) Denaturing agarose gel electrophoresis, of the RNA transcription products from J3 cDNA. Lane 1. 5 ul of J3 uncapped transcript. Lane 2. 10 ul of J3 uncapped transcript. Lane 3. 1 kb NEB RNA ladder. (b) In vitro Transcription. Lane 1. J3 uncapped transcript. Lane 2. X1S mutant uncapped transcript. Lane 3. 1A sub genomic uncapped transcript. Lane 4. 1A sub genomic uncapped transcript.

Transcription of the clones J3 (genomic), X1 (mutant) and 1A (sub genomic) resulted in bands of approximately 7.5 kb for both J3 and X1, and a band of approximately 2.5 kb for 1A. An additional band of approximately 3.5 kb was observed for the J3 and X1 reactions, which is presumably due to premature termination of transcription (Figure 1).

## Discussion

Carlow virus (Hu/NoV/GII/Carlow/2002/Ire) genome is 7559 nts in length, excluding the 3'-end poly A tail and represents the first norovirus isolate from Ireland to be sequenced. Alignments of the entire genome of Carlow virus revealed 99% identity to the Norovirus GII Genotype 4 variant members HU/NoV/Farmington Hills/2002/USA [GenBank: AY502023] and the United Kingdom strain B4S6 [GenBank: AY587985]. Farmington Hills was associated with 64% of all cruise ship outbreaks in the United States, whereas its UK counterpart was implicated in 100% of 22 outbreaks in Oxfordshire during 2002 and 2003 [20,8].

Genomes submitted prior to 2002 appeared to exhibit less identity with Carlow virus such as the prototype GII.4 member Lordsdale virus [21] [GenBank: X86557], which displayed 90% identity over the entire genome. 'A novel norovirus strain is defined as having 90% or less identity at the nucleotide level with published sequences' [22]. In addition, Carlow virus contains the six nucleotide motif ('AATCTG' at nt 4546–4552) not present in any genogroup II genotype 4 noroviruses prior to 2002, and is indicative of the norovirus variant genotype [23]. Therefore, Carlow virus is a member of the Farmington Hills variant cluster of Genogroup II genotype 4 noroviruses.

DNAstar EDITseq can predict the potential charge of a protein. The most significant changes in this predicted charge was for the polyprotein of Carlow virus with a charge of 9.1 at pH 7 compared to Lordsdale virus, which has a charge of 7.3 at pH 7. This change in charge is attributable to a number of amino acid changes between the two proteins. Comparing proteins prior to and from 2002, a pattern of amino acids, which, appeared to be conserved prior to 2002 were substituted for alternative amino acids, which, appear to be conserved after 2002. This phenomenon was evident for all three ORFs. Most of these substitutions were with amino acids that were conserved with regard to charge and/or structure. However, for the majority of polyprotein sequences aligned, 6 amino acid substitutions, (before 2002) A, A, Q, Q, P, K change to (after 2002) T, T, K, E, S and T, respectively (a.as 782, 791, 1045, 1291, 1456 and 1480) resulting in the potential change in charge and/or structure of the polyprotein.

**Figure 2:**
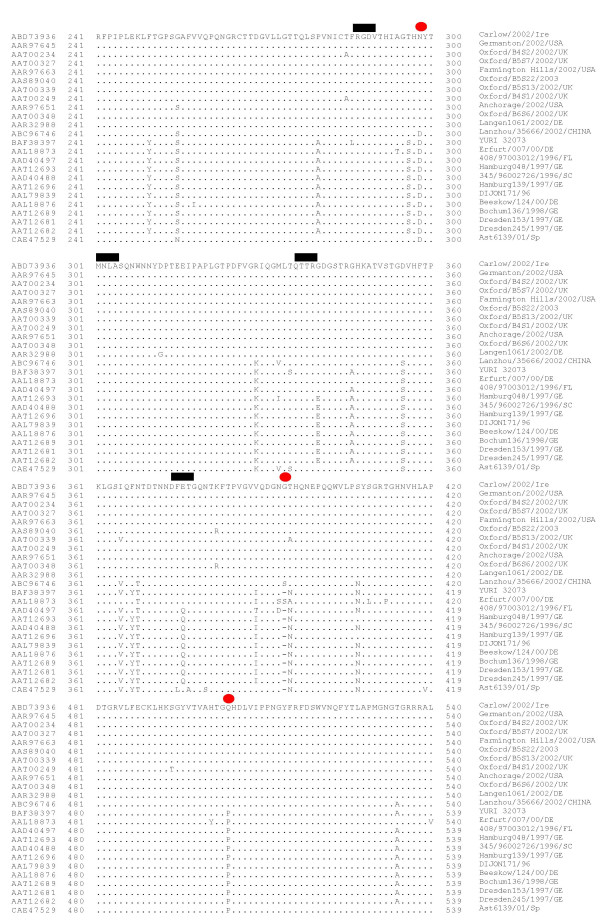
The alignment of amino acids 241–419 and 481–540 of the potential capsid protein of Carlow virus, with the corresponding proteins of 12 post and 11 pre 2002 viruses. The red circle indicates amino acids conserved prior to 2002 which have been substituted for an amino which are conserved in the majority of viruses after 2002 (exception Lanzhou/35666/2002/china and Yuri 32073/Japan). These changes are to un-conserved amino acids, acidic to basic for example. The thick black line shows the RGD motif and 3 additional motifs responsible for strain specific binding of the human blood group antigens [3].

In the majority of VP1 sequences, D_298 _and N_394 _were replaced with N and G after 2002. These amino acids were of interest as they were located within the predicted P2 domain of Carlow Virus. Based on sequence homologies, the P2 domain of Carlow virus contains the NGR motif (required for receptor binding) followed by a binding pocket determined by Tan et al., 2003 [3]. This pocket consists of an RGD-like motif (site I) and three additional sites II, III and IV located between amino acids 268–418 (Figure 2). This pocket is considered to be responsible for the binding of the human blood group antigens [3]. Notably, site IV mutates from DFQ (before 2002) to DFE (374–377, after 2002) in a significant number of sequences aligned (Figure 2). This mutation also occurred in Genogroup II genotype 4 noroviruses in Japan between 1999 and 2004 [24].

Whether or not any of these amino acids have a role in altering binding specificity would require mutational analysis of the capsid protein, for use in, in vitro binding assays.

Vp2 is a minor structural protein and has been shown to stabilise the VP1 (VLP) particle and protect it from protease degradation in, in vitro studies [25]. In addition, it has a highly basic charge, which has resulted in the suggestion of a role in RNA binding [26]. The greatest degree of divergence was observed for VP2 of Carlow virus when comparing sequences in Genbank (98% to Farmington Hills compared to 99% for the polyprotein and VP1 and only 86% identity was observed to VP2 of Lordsdale virus [GenBank: CAA60256]). Interestingly, the amino acid changes that occurred in VP2 of Carlow virus were primarily conserved with regard to charge. One substitution, K to E at amino acid 80 is of interest as it results in a significant change from a basic to an acidic residue. Mutational analysis would be necessary to elucidate the impact of this amino acid alteration.

Transcription of the clones J3 (genomic), X1 (mutant) and 1A (sub genomic) resulted in bands of approximately 7.5 kb for both J3 and X1, and a band of approximately 2.5 kb for 1A. An additional band of approximately 3.5 kb was observed for the J3 and X1 reactions, which is presumably due to premature termination of transcription. Premature termination is observed for the transcription reaction of the feline calicivirus vaccine strain 2024 from the cDNA pIK12 clone [27].

## Conclusion

In conclusion, Carlow virus is a member of the Genogroup II, genotype 4 variant cluster of noroviruses, of which Farmington Hills is the prototype.

## Methods

### Noroviruses

70 stool samples were obtained from five different suspected Norovirus outbreaks from hospital settings in the south eastern region of Ireland in the 2002–2003 season. Random samples were taken from symptomatic individuals suspected to harbour Norovirus and stored at 4°C. Our laboratory was notified to collect samples which were then transported in sealed containers at 4°C.

### RNA extraction and cDNA synthesis

Upon receipt of the samples, a 10% suspension was made in DMEM (Sigma), vortexed briefly and clarified by centrifugation at 10,000 rpm for 10 min. RNA was extracted from 140 μl of supernatant using the Viral RNA mini kit (Qiagen) and eluted in 50 μl of AVE buffer according to the manufacturers instructions. The RNA was treated with 2 μl DNase I (Ambion) and stored at -80°C.

### Standard Reverse Transcription

Reverse transcription was performed with 5 μl of RNA template utilising SuperScript ™ II reverse transcriptase (Invitrogen) and random hexamer primers (Roche) as outlined in the manufacturers instructions.

### Assembling the full length clone by Polymerase Chain Reaction (PCR)

5 μl of sample 13 cDNA was used as a template for amplification in PCR. Primers JV12 and SM31 [28], which are specific for the polymerase gene of Norovirus were used to amplify a 333 nt fragment. This fragment was cloned into the Topo II dual vector from Invitrogen, transformed into Top10 cells (Invitrogen) in the presence of Ampicillin (Sigma) 100 μg/ml, sequenced (MWG Biotech) and was designated **Pol9.**

### Screening

Screening primers COG2F (Table 1, [19]) and a reverse primer G2NVR designed in our laboratory, (Table 1) were used to generate a 384 nt PCR product from the ORF1/ORF2 overlap region. 28 from 70 stool samples obtained from 2002 generated a product utilising these primers. Six of these sample products were sequenced (MWG Biotech) and found to be Genogroup II genotype 4. One such sample product (sample 13) was cloned into pCR2.1 vector (Invitrogen) to generate **caps2 **and **5 **clones.

**To generate the 3' half of the genome**, the reverse transcription reaction was performed utlising 10 μl of sample 13 RNA, 50 pmoles of TX30SXN [29] and 20 mM dNTPS according to the manufacturers instructions using 1 ul of Thermoscript (Invitrogen). PCR was employed using primers ORF3minusdegerate (Table 1) and COG2F (Table 1, [19]) in a 50 μl volume utilising 1 μl of Platinum^® ^Taq DNA Polymerase High Fidelity (Invitrogen) as outlined by the manufacturer.

**Figure 3:**
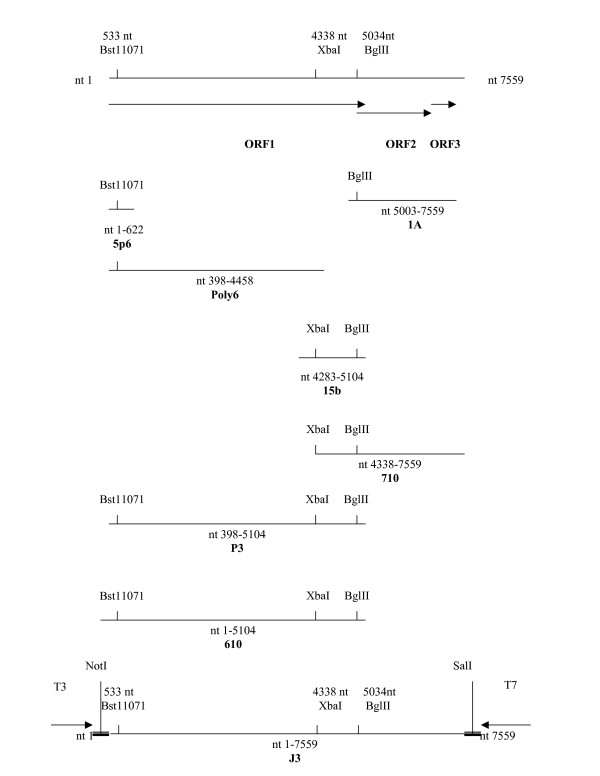
A schematic representation of the genomic organisation of Carlow virus. ORF's 1, 2 and 3 are indicated by arrows. Restriction sites and the clones necessary for generation of the genome are shown. Thickened lines indicate vector sequences whereas arrows represent the T3 and T7 phage promoters.

A 2.9 kb fragment was cloned into the *Eco*RV (Roche) digested pBlueScript II SK+ vector (Stratagene). The subsequent clone assigned **1A **contained nts 5003 to 7559 of Carlow Virus (Figure 3). This band was as a result of carry over of the excess TX30SXN [29] utilised in the reverse transcription and COG2F primer [19].

**To generate the 5' half of the genome**, reverse transcription was approached as for the 3' end with the following exceptions; 5 μl of sample 13 RNA and 50 pmoles of 4440 minus (Table 1) were utilised. PCR was performed using 5 μl of sample 13 cDNA, 50 pmoles of primers 430F and 4440 nest (Table 1) as previously described with the inclusion of a final concentration of 1% Dimethylsulfoxide (DMSO) (Sigma).

The resultant 4 kb band was cloned into the *Eco*RV (Roche) digested pBlueScript II SK+ vector. The subsequent clone designated **Poly6 **harboured nt 398 to 4458 of Carlow Virus (Figure 3).

In order to fill the gap between nt 4458 to 5003, 2 μl of the cDNA generated from the 3' end transcription, was utilised as a template in addition to 50 pmoles of 5090 reverse, 4290 forward primers (Table 1) and 1 μl Platinum^® ^Taq DNA Polymerase High Fidelity in a PCR reaction according to the manufacturers instructions. The 827 nt fragment was cloned into the *Eco*RV (Roche) digested pBlueScript II SK+ and labelled **15b**. This clone contains nt 4283 to 5104 of Carlow virus (Figure 3).

**The immediate 5' end **of Carlow Virus was determined utilising cDNA generated from a standard reverse transcription reaction, utilising random hexamer primers. The PCR reaction utilised 1 μl of 50 pmol/μl of NLV1S25, NEW RACE primers, (Table 1) and Taq polymerase (FINNZYME Oy) as directed by the manufacturer. A 622 nt fragment was generated and cloned and assigned **5p6**. Nucleotide (nt) 1 – 24 corresponds to the primer, nt 25 – 622 corresponds to Carlow virus (Figure 3) (note: Numerous attempts at 5' RACE (Invitrogen) to generate the immediate 5' end did not yield sequence between nt 1 and 36).

The purpose of the following cloning experiments was to link the four clones **5p6**, **Poly6**, **15b **and **1A **by employing restriction enzymes rather than subjecting the template to any additional rounds of PCR.

The 819 nucleotide fragment of **15b **was excised with *Xba*I (Roche) and cloned into *Xba*I digested **Poly6 **to yield pBlueScript II SK+ (Stratagene) harbouring nt 398–5104 of Carlow virus. This construct was sequenced to ensure correct alignment, and was designated **P3 **(Figure 3, nt 398–5104 of Carlow virus).

**710 **is the 696 nt *Xba*I-*Bgl*II (nt 4338–5034) fragment of **15b **cloned into the 5.8 kb *Bgl*II-*Xba*I (Roche) **1A **fragment and was confirmed by sequencing (Figure 3, nt 4338–7559 of Carlow virus).

Clone **610 **is the 639 nt *Xho*I-*Bst*11071 (Roche) fragment of **5p6 **cloned into the *Sal*I-*Bst*11071 (Roche) digested **P3 **clone and confirmed by sequencing (Figure 3, nt 1–5104 of Carlow virus).

The 4.3 kb *Not*I-*Xba*I (Roche) fragment of **610 **(nt 1–4338) was cloned into the 6.3 kb *Xba*I -*Not*I (nt 4338–7559) fragment of **710, **to yield **J3. **This clone was sequenced to ensure the correct alignment of the entire 7759 nucleotide Carlow virus genome (Figure 3, nt 1–7559).

### The polymerase mutant genome denoted X1

**X1 **was generated by *Xba*I digestion of J3, followed by end filling using Klenow enzyme (Promega) in the presence of 1.1 μM dNTPs as outlined by the manufacturer. Sequence analysis confirmed the insertion of the four nucleotides CTAG within the polymerase gene at nt 4346 of Carlow virus [GenBank: DQ415279]. This mutation results in a stop codon in the predicted amino acid sequence of the Carlow virus non structural polyprotein at amino acid 1454.

### In vitro transcriptions

pBlueScript II SK+ (Stratagene) possesses both a T3 and T7 phage promoter either side of the multiple cloning region. The clones J3, X1 and 1A all have a T3 promoter upstream of the cloned viral cDNA and a T7 promoter downstream. The poly A tail is followed by a *Sal*I restriction site (Figure 3). The 5' end is preceded by an *Not*I site (Figure 3).

Capped and uncapped RNA transcripts were produced utilising 1 μg of *Sal*I digested J3, X1 and 1A cDNAs respectively, in standard in vitro transcription reactions, in a 120 μl volume, utilising the Ambion Megascript^® ^T3 kit (Ambion) and manual. The samples were incubated at 37°C for 6 hours, treated with Dnase I (Ambion) for 1 hour at 37°C. The resultant transcripts were treated with 3 μl of 0.5 M EDTA and cleaned using a High Pure PCR Purification Kit (Roche). The RNA was resuspended in 50 μl of RNase free, DNase free H_2_O. The concentration of plus strand RNA was determined by spectrophotometry. The quality of RNA was determined by denaturing gel electrophoresis.

## Competing interests

Department of Microbiology, University College Cork

Irish Government

## Authors' contributions

Dr. Karen Kearney is the corresponding and main contributing author of this manuscript. Screening results were provided by John Menton. Supervision and final review was contributed by Dr. John Morgan. All authors have read and approved this manuscript.
